# Breast cancer Ki-67 expression prediction by digital breast tomosynthesis radiomics features

**DOI:** 10.1186/s41747-019-0117-2

**Published:** 2019-08-14

**Authors:** Alberto Stefano Tagliafico, Bianca Bignotti, Federica Rossi, Joao Matos, Massimo Calabrese, Francesca Valdora, Nehmat Houssami

**Affiliations:** 1 Department of Health Sciences (DISSAL), Radiology Section, University of Genoa, Genoa, Italy; 2 Emergency Radiology, IRCCS Policlinico San Martino, Genoa, Italy; 3 Sydney School of Public Health, Sydney Medical School, University of Sydney, Sydney, Australia

**Keywords:** Breast neoplasms, Cell proliferation, Ki-67 expression, Mammography, Radiomics

## Abstract

**Background:**

To investigate whether quantitative radiomic features extracted from digital breast tomosynthesis (DBT) are associated with Ki-67 expression of breast cancer.

**Materials and methods:**

This is a prospective ethically approved study of 70 women diagnosed with invasive breast cancer in 2018, including 40 low Ki-67 expression (Ki-67 proliferation index <14%) cases and 30 high Ki-67 expression (Ki-67 proliferation index ≥ 14%) cases. A set of 106 quantitative radiomic features, including morphological, grey/scale statistics, and texture features, were extracted from DBT images. After applying least absolute shrinkage and selection operator (LASSO) method to select the most predictive features set for the classifiers, low *versus* high Ki-67 expression was evaluated by the area under the curve (AUC) at receiver operating characteristic analysis. Correlation coefficient was calculated for the most significant features.

**Results:**

A combination of five features yielded AUC of up to 0.698. The five most predictive features (sphericity, autocorrelation, interquartile range, robust mean absolute deviation, and short-run high grey-level emphasis) showed a statistical significance (*p* ≤ 0.001) in the classification. Thirty-four features were significantly (*p* ≤ 0.001) correlated with Ki-67, and five of these had a correlation coefficient of > 0.5.

**Conclusion:**

The present study showed that quantitative radiomic imaging features of breast tumour extracted from DBT images are associated with breast cancer Ki-67 expression. Larger studies are needed in order to further evaluate these findings.

## Key points

- The association between quantitative radiomic features from digital breast tomosynthesis and Ki-67 expression of breast cancer was investigated.
- A combination of five radiomic features yielded an area under the curve at receiver operating characteristics analysis of 0.676 to for high *versus* low Ki-67 expression.
- Thirty-four features were significantly correlated with Ki-67 expression.

## Background

The proliferation marker Ki-67 is an independent predictive and prognostic factor for breast cancer patients [1]. Although several attempts are ongoing toward personalised medicine with complex approaches to find the best biomarkers for an individual patient, Ki-67 has been shown to successfully guide the optimal treatment for each subject [2, 3]. Indeed, whilst a poor prognosis in breast cancer patients is usually associated with high Ki-67 expression, patients with high Ki-67 expression are likely to respond better to chemotherapy. The first evaluation of Ki-67 on breast cancer patients is based on immunohistochemistry obtained often on vacuum/assisted breast biopsy (VABB) or core biopsy. However, tumoural heterogeneity and limited extent of the retrieved samples explain why the samples might be not representative of the entire tumour bulk and hence of its biology.

In recent years, the increasing use of radiomics in medical imaging to identify biomarkers for prognosis and therapy monitoring relies on the reasonable assumption that tumour characteristics, in particular its heterogeneity, can be assessed directly from a clinical medical image (which captures the entire tumour) rather than from a relatively limited tissue sample. Radiomics refers to the extraction and analysis of large amount of quantitative imaging features from medical images and has been extensively discussed in recent literature [4, 5]. In an exploratory study, it has been proposed that quantitative imaging features extracted from dynamic contrast-enhanced magnetic resonance imaging (DCE-MRI) are associated with breast cancer Ki-67 expression [6]. However, women with breast cancer are more likely to have DBT available instead of MRI which is not performed routinely in all breast cancer cases. Therefore, the aim of this study was to investigate whether quantitative radiomic features extracted from DBT images are associated with Ki-67 expression of breast cancer.

## Methods

### Patients

This prospective study was approved by the institutional review board, and written informed consent requirement was acquired (CER009/2018). The study cohort included 70 patients diagnosed with invasive breast cancer (confirmed at histopathology after surgery) from January to December 2018. Patient characteristics are reported in Table 1.The patient group included cancers with 40 low Ki-67 expression (Ki-67 proliferation index < 14%) and 30 high Ki-67 expression (Ki-67 proliferation index ≥ 14%). The cut-off value of 14% for Ki-67 proliferation index was selected to be consistent with recent literature on MRI [6].

**Table 1:** Patients and tumour characteristics

Characteristic	Mean (range)	Number of patients
Age	62 (26–86) years	70
Histological subtype		
Invasive ductal carcinoma		52
Infiltrating lobular carcinoma		8
Other types		10
Histological grade (Nottingham’s scale)		
High (G3)		16
Intermediate (G2)		28
Low (G1)		26
Tumour size	15 (4–55) mm	
< 10 mm		12
10–19 mm		45
20–29 mm		5
≥ 30 mm		8
Nodal status		
Negative		48
Positive		16
Unknown		6

### Digital breast tomosynthesis

Tomosynthesis acquisitions were performed with reconstructed synthesised two-dimensional images, using a commercially available equipment (Hologic, Selenia Dimensions, Bedford, MA, USA). Radiomic analysis was performed as previously done on DBT images by Tagliafico et al. [7] who firstly applied and reported a radiomic approach to DBT to differentiate normal from malignant breast tissue in patients with dense breasts [5].

### Image analysis

**Fig. 1:**
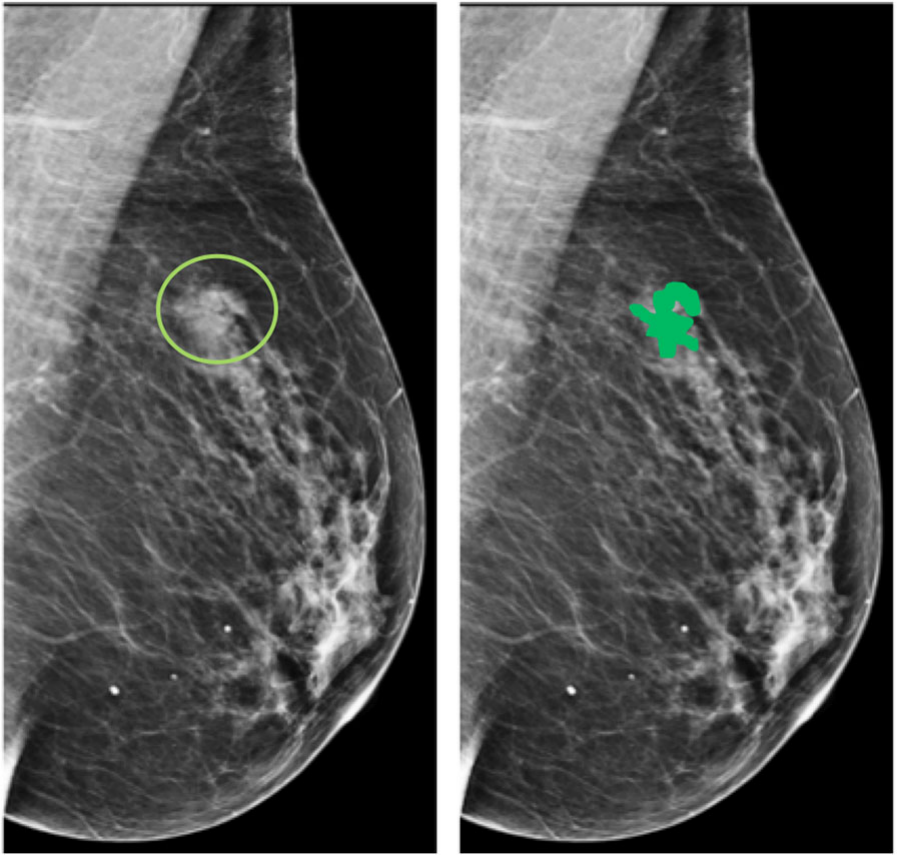
Medio-lateral oblique view (two-dimensional image). On the left, the circle highlights the suspicious spiculated lesion. On the right, the region of interest (coloured in green) defined by the reader. The region of interest was then transferred to the central tomo slices using a software and adjusted if necessary

Radiomic analysis was performed on all DBT images; ROI positioned on 2D images were transferred to the central tomo slices using software (Osirix and 3D Slicer 4.7), within manually selected regions of interest (ROIs) including all DBT areas subjectively attributed to malignant tissue. ROIs were placed by two researchers (AT and FV) with expertise in quantitative image analysis (9 and 5 years of experience, respectively). ROI tracing on the single slice of the central digital breast tomosynthesis projection images was adjusted manually, if necessary [7]. An example is shown in Fig. 1. From DBT images, we extracted 106 features using an open-source software platform for medical image informatics, image processing, and three-dimensional visualisation (3D Slicer 4.7; www.slicer.org) built over two decades through support from the National Institutes of Health and a worldwide developer community [8]. Details of the mathematical notations and the computation of these texture features have been previously published [9]. All these features were normalised to a standard range before being used in a statistical software.

### Statistical analysis

Least absolute shrinkage and selection operator (LASSO) method [10] to select the most predictive feature set for the classifiers was used, and low *versus* high Ki-67 expression prediction was evaluated by receiver operating characteristic (ROC) analysis. For significant features, a multivariate analysis and multiregression correlation analysis and the results in terms of true positive and false positive for the significant features were done. Accuracy was measured using ROC analysis to estimate the area under the curve (AUC). Radiomic features that significantly differentiated low *versus* high Ki-67 expression, based on the mean value in each group, were considered if AUC was > 0.6. Ninety-five percent confidence intervals (95% CIs) were calculated. Using a statistical software, *p* values below 0.05 were considered statistically significant. Correlation analysis and univariate linear regression were performed to determine the association between the individual radiomic features and the Ki-67 expression. Statistical tests were done using a statistical software (STATA MP, StataCorp, 4905 Lakeway Dr, College Station, TX, USA, and MedCalc).

## Results

**Fig. 2:**
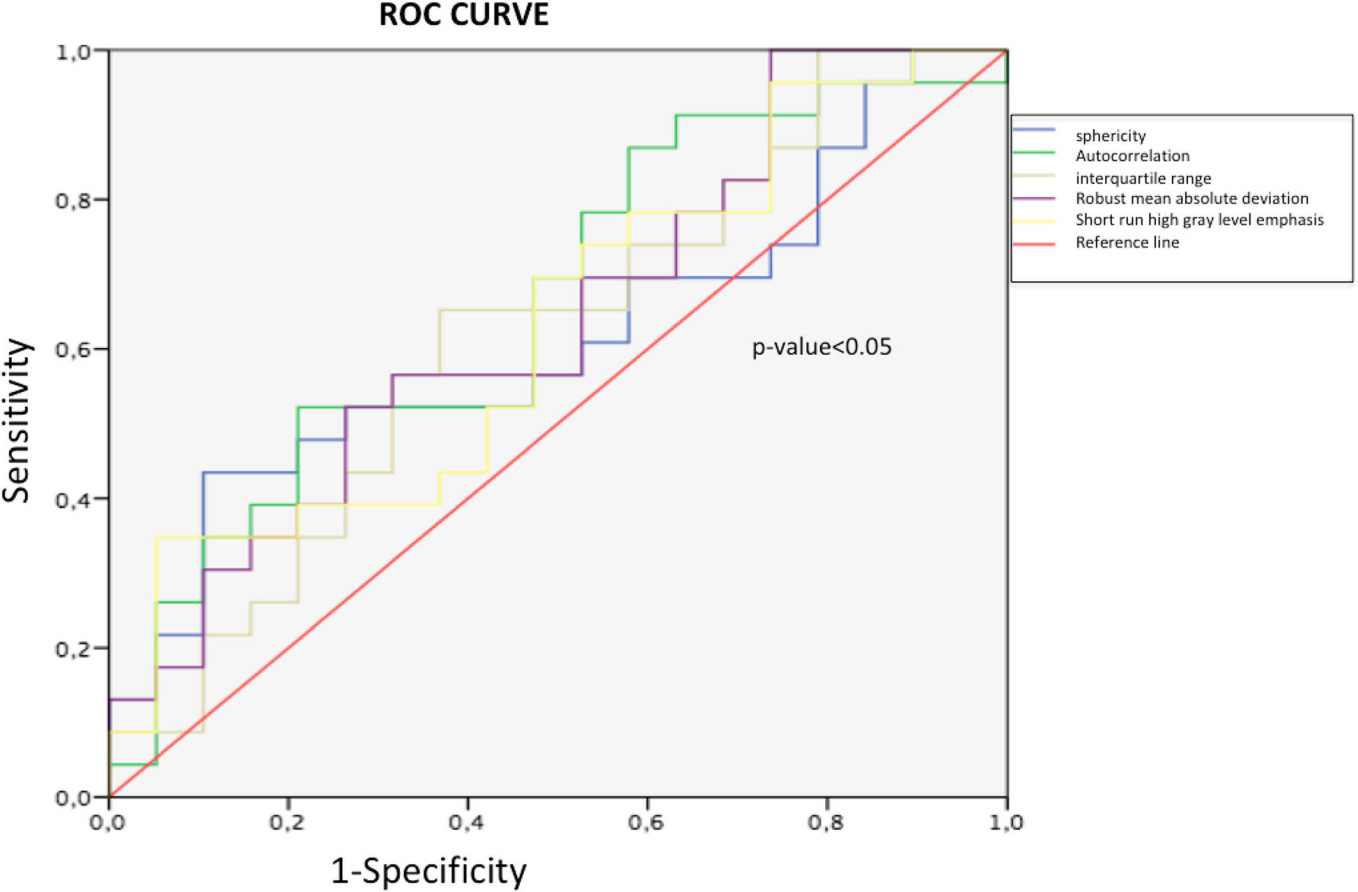
Receiver operating characteristic curve of feature parameters differetiating between low and high Ki-67 expression on digtal breast tomosynthesis. Features were included if the area under the curve was > 0.6 in association with statistical significance

There was no significant difference in age between the low Ki-67 group and the high Ki-67 group (*p* = 0.523). On the basis of the multivariate logistic regression analysis, the clinical factors with *p* < 0.05 were used to build the clinical model. Calibration was plotted to explore the predictive accuracy by bootstrapping with 1000 resamples. The list of radiomic features with significant *p* values and AUC > 0.6 for discriminating low from high Ki-67 expression lesions is reported in Table 2 and Fig. 2.

**Table 2:** Values of feature parameters differetiating between low and high Ki-67 expression on digital breast tomosynthesis

Feature	Feature class	AUC	*p* value	Lower 95% CI	Upper 95% CI
Sphericity	Shape	0.613	0.007	0.462	0.764
Autocorrelation	Grey level co-occurrence matrix	0.625	0.002	0.474	0.776
Interquartile range	First order	0.633	0.003	0.481	0.784
Robust mean absolute deviation	First order	0.641	0.009	0.487	0.795
Short-run high grey-level emphasis	Grey level run length matrix	0.676	0.001	0.534	0.818

Features were included if the area under the curve (AUC) was > 0.6 with a *p* < 0.05. *CI* Confidence interval

In terms of the most significant features selected by the LASSO method to be correlated with Ki-67, 34 features were significantly (*p* ≤ 0.001) correlated with Ki-67 and 5 of these had a correlation coefficient of > 0.5. These five features were median, total energy, different average, tenth percentile among first order features, and grey level co-occurrence matrix (GLCM) (Table 3). Sphericity is a morphological feature describing the three-dimensional size and shape of the tumour, whereas autocorrelation is a measure of the magnitude of the fineness and coarseness of texture. Interquartile range and robust mean absolute deviation are first-order features representing the mean distance of all intensity values from the mean intensity calculated on the subset of image array with grey levels in-between, or equal to the 10th and 90th percentile representing intra-tumoural heterogeneity. Short-run high grey-level emphasis is a grey level run length matrix (GLRLM) feature and increases when the texture is dominated by short runs with high intensity levels likely also reflecting intra-tumoural heterogeneity.

**Table 3:** Values of the five features parameters with a correlation coefficient > 0.5 and *p* < 0.001

Feature name	Feature class	Correlation coefficient	*p* value
Median	First Order	0.536	0.001
Total energy	First Order	0.622	0.001
Different average	First Order	0.501	0.001
10th percentile	First Order	0.658	0.001
Contrast	Grey level co-occurrence matrix	0.616	0.001

Table 4 reports the performances of the best five features of Table 2.

**Table 4:** Performance metrics of the five features with area under the curve > 0.6 to differentiate low and high Ki-67 expression on digital breast tomosynthesis

Feature	Feature class	Accuracy	Sensitivity	Specificity
Sphericity	Shape	0.656	0.645	0.673
Autocorrelation	Grey level co-occurrence matrix	0.677	0.682	0.653
Interquartile range	First order	0.653	0.661	0.631
Robust mean absolute deviation	First order	0.676	0.689	0.631
Short-run high grey-level emphasis	Grey level run length matrix	0.698	0.742	0.534

## Discussion

The present study used DBT images to apply a radiomic approach aiming to assess if an association between breast cancer Ki-67 expression and radiomic features exists. Our findings show that after applying methods to select the most predictive features to differentiate low *versus* high Ki-67 expression, it is possible to identify several features with ROC-AUC values from 0.653 to 0.698 to differentiate patients with low *versus* high Ki-67 expression. We could speculate that with refinements in textural analysis techniques and increased reproducibility radiomic analysis, it may be possible to obtain an estimation of Ki-67 expression directly from DBT images even before biopsy, estimating the activity of the tumour (namely cell proliferation) directly from DBT images obtained at diagnosis. This hypothesis was already developed by a previous pilot study where quantitative radiomic imaging features of breast tumour extracted from DCE-MRI were associated with breast cancer Ki-67 expression [6]. In the present study, we showed that using an imaging modality such as DBT, used in breast cancer screening and diagnosis and more widely available than MRI, it is possible to find an association between quantitative radiomic imaging features and Ki-67 expression. The data reported in the present study are encouraging because they support the hypothesis that radiomic features could predict Ki-67 expression of breast cancer (and hence an element of tumour biology) in a non-invasive manner.

We selected features that simultaneously resulted in an AUC > 0.6 with a minimum *p* value of 0.05 in differentiating between low and high Ki-67 expression on DBT. This allowed us to identify five features with these requirements, whereas a previous study that was based on DCE-MRI found 13 features associated with Ki-67 expression (similarly meeting *p* < 0.05 and AUC > 0.6 criteria) [6]. As a whole, the meaning of these features could be that high Ki-67-expressed lesions are more likely to be inherently heterogeneous. The five DBT-based features identified in this study differ from the DCE-MRI-based features, but they reflect intra-tumoural heterogeneity as well. Moreover, the type of information contained in MRI *versus* DBT images is different due to the different biophysical characteristics of the two imaging modalities.

Our study suggests that tumoural biological aggressiveness related to high Ki-67 expression can be evaluated on DBT, avoiding the need of contrast media injection which is used for MRI. In this study, we did not find features with AUC higher than 0.7; therefore, it seems possible that radiomic features on DBT do not strongly discriminate low and high Ki-67-expressing breast cancers and further evaluation in larger studies is needed. However, future studies could examine the added value of radiomic evaluation (added to other prognostic factors) in tumoural aggressiveness assessment. A previous study [7] on radiomics and DBT performed in women with dense breast found a correlation between radiomic features and tumour size and oestrogen receptors: three radiomic features (energy, entropy, and dissimilarity) correlated with tumour size and entropy correlated with oestrogen receptor status. This study supports the use of radiomics to assess tumoural characteristics on breast cancer patients on digital breast tomosynthesis in further studies based on larger datasets to extend these exploratory data.

This study has several limitations. The first limitation is that images are from a single vendor and acquired at a single institution; therefore, a multicentric study would be important to assess if these results are valid on a larger scale and could be generalised. The second limitation is that tumour lesions were manually segmented by human readers and reproducibility was not assessed. As a major limitation, we acknowledge that there are no data on reproducibility in this study, although the methods of the present paper were already tested in our previous paper (intra-observer agreement of 0.78) [7].

The third limitation is that we have few published data regarding DBT and radiomics, and this is one of the first studies exploring associations between radiomic features and Ki-67 expression on DBT; therefore, comparison with previously published data is limited.

However, the results of this study are not sufficient to make decisions in clinical practice because of the relatively limited AUC values found for the features and because of these results need to be replicated by other researchers. In addition, the study design was not intended to create an algorithm or a radiomic nomogram to differentiate low from high Ki-67 expression. We acknowledge the exploratory nature of the present research.

However, this study could be used for future study not only as a proof-of concept investigation but also as a reference for comparisons.

In terms of strengths, whilst noting this as an exploratory study, it is nonetheless the first study to use data from images prospectively acquired with the same technique reducing biases due to image acquisition geometry and reconstruction algorithm typical of DBT prototypes. Also, we used a freely available software enhancing the ability to replicate the research by other independent groups.

In conclusion, this study showed that some quantitative imaging features extracted from DBT acquired in clinical practice are associated with breast cancer Ki-67 expression. Our findings can inform new studies on DBT-based radiomics and breast cancer biology.

## Data Availability

The datasets used and/or analysed during the current study are available from the corresponding author on reasonable request.

## Abbreviations

95% CIs: Ninety-five percent confidence intervals
AUC: Area under the curve
DBT: Digital breast tomosynthesis
DCE-MRI: Dynamic contrast-enhanced magnetic resonance imaging
GLCM: Grey level co-occurrence matrix
LASSO: Least absolute shrinkage and selection operator
ROC: Receiver operating characteristic
